# A portable transistor immunosensor for fast identification of porcine epidemic diarrhea virus

**DOI:** 10.1186/s12951-024-02440-5

**Published:** 2024-05-12

**Authors:** Xiao Hu, Mengjia Zhang, Yiwei Liu, Yu-Tao Li, Wentao Li, Tingxian Li, Jiahao Li, Xueqian Xiao, Qigai He, Zhi-Yong Zhang, Guo-Jun Zhang

**Affiliations:** 1 School of Laboratory Medicine, Hubei University of Chinese Medicine, 16 Huangjia Lake West Road, Wuhan, 430065 P.R. China; 2 Department of Pharmacy, Renmin Hospital, Hubei University of Medicine, Shiyan, Hubei 442000 P.R. China; 3 National Key Laboratory of Agricultural Microbiology, College of Veterinary Medicine, Huazhong Agricultural University, Wuhan, 430070 P. R. China; 4 Key Laboratory of Prevention & Control for African Swine Fever and Other Major Pig Diseases, Key Laboratory of Development of Veterinary Diagnostic Products, Ministry of Agriculture and Rural Affairs, Wuhan, 430070 P. R. China; 5 Hunan Institute of Advanced Sensing and Information Technology, Xiangtan University, Hunan, 411105 P. R. China; 6 Key Laboratory for the Physics and Chemistry of Nanodevices and Center for Carbon-based Electronics, School of Electronics, Peking University, Beijing, 100871 P. R. China; 7 Hubei Shizhen Laboratory, Wuhan, Hubei 430065 P.R. China

**Keywords:** Porcine epidemic diarrhea virus, Monoclonal antibody, Immunosensor, Spike protein, Carbon nanotubes, Field-effect transistor

## Abstract

Widespread distribution of porcine epidemic diarrhea virus (PEDV) has led to catastrophic losses to the global pig farming industry. As a result, there is an urgent need for rapid, sensitive and accurate tests for PEDV to enable timely and effective interventions. In the present study, we develop and validate a floating gate carbon nanotubes field-effect transistor (FG CNT-FET)-based portable immunosensor for rapid identification of PEDV in a sensitive and accurate manner. To improve the affinity, a unique PEDV spike protein-specific monoclonal antibody is prepared by purification, and subsequently modified on FG CNT-FET sensor to recognize PEDV. The developed FET biosensor enables highly sensitive detection (LoD: 8.1 fg/mL and 10^0.14^ TCID_50_/mL for recombinant spike proteins and PEDV, respectively), as well as satisfactory specificity. Notably, an integrated portable platform consisting of a pluggable FG CNT-FET chip and a portable device can discriminate PEDV positive from negative samples and even identify PEDV and porcine deltacoronavirus within 1 min with 100% accuracy. The portable sensing platform offers the capability to quickly, sensitively and accurately identify PEDV, which further points to a possibility of point of care (POC) applications of large-scale surveillance in pig breeding facilities.

**Supplementary Information:**

The online version contains supplementary material available at 10.1186/s12951-024-02440-5.

## Introduction

Porcine epidemic diarrhea (PED), an acute, highly contagious intestinal infectious disease caused by porcine epidemic diarrhea virus (PEDV), is characterized by severe watery diarrhea, vomiting and dehydration in newborn piglets [1–3]. Since its first report in 1977 in Belgium, PEDV has spread rapidly to Japan, China, the United States and other countries [4–8]. In late 2010, a highly virulent PEDV was identified and found to be prevalent in China, with 80% - 100% morbidity and 50% - 90% mortality in suckling piglets, which has caused devastating economic losses in the pig industry [9–11]. Generally, infection with PEDV and other porcine intestinal coronaviruses, such as transmissible gastroenteritis virus (TGEV) and porcine deltacoronavirus (PDCoV) could also induce similar symptoms. However, PEDV infection is of most severe and induces the highest mortality rate [3, 12]. Therefore, rapid and accurate identification is essential for timely interruption of PEDV transmission, preventing major outbreaks.

Currently, the detection of PEDV is implemented by examination of the viral proteins, genome, or antibodies [13–16]. Polymerase chain reaction (PCR)-based nucleic acid testing has high sensitivity and specificity in the diagnosis of viral infections, but also has drawbacks of requiring the design of complex primers and probes, expensive instrumentation, trained operators, and long “sample to answer” time [17, 18]. PEDV antibodies can only be detected around 10  -  14 days post infection and are henceforth not appropriate for fast detection. In contrast, viral antigen testing can be performed quickly by eliminating the steps of RNA extraction, reverse transcription and amplification. To date, antigen detection for PEDV is usually performed by enzyme-linked immunosorbent assays (ELISA) [13, 19, 20], lateral flow assays (LFA) [21–23] or electrochemical assays [2, 24–26]. Implementation of those methods normally requires complicated procedures or long-term analysis times (ELISA), or suffer from low sensitivity (LFA and electrochemical). Therefore, it is imperative to establish a ultrasensitive and on-site method for PEDV detection.

Field effect transistor (FET), a highly encouraged biosensing platform, is characterized by its excellent sensitivity, label-free and real-time detection, low cost, and easy integration [27–29]. Currently, FET-based biosensors are increasingly applied to the detection of various biomarkers [30–34]. Among various nanomaterials, carbon nanotubes (CNTs) have been considered ideal channel materials for FET biosensors due to their ultra-thin thickness, superior electrical properties and good biocompatibility [35–38]. In a previous study, we constructed a plug-and-play floating gate CNT field-effect transistors (FG CNT-FET) biochip integrated with a smart portable reader [39]. It was combined with a modified CRISPR/Cas12a system that introduced a G-triple-stranded structural reporter to achieve ultrasensitive and point-of-care testing of cardiac troponin I, showing promising applications. Nevertheless, the application of FG CNT-FET sensor we developed for PEDV identification has not yet been demonstrated.

The single-stranded positive-stranded RNA genome of PEDV is approximately 28 kb in length and encodes 4 structural proteins: spike protein (S-protein), membrane protein, small envelope protein and nucleocapsid protein (N-protein) [12]. Most recent studies centered on capturing PEDV using antibodies that targeted the N-protein, which is proven to be challenging as the N-protein is located inside the virion [19, 20, 40]. S-protein is a fibrillar glycoprotein located on the surface of viral particles and plays an important role in regulating receptor-mediated viral invasion as well as inducing production of neutralizing antibodies [41–43]. Therefore, it is essential to develop *de novo* antibodies targeting the S-protein to directly capture virus particles. Typically derived from a clonal expansion of conventional or genetically engineered hybridomas, monoclonal antibodies (mAbs) have broad usages under different situations including immunotherapy and diagnosis. Due to the advances in mAb research, mass production and tailor-made adjustments are currently approachable, while their robust, highly specific affinities against their target epitopes are often utilized for the development of immune-sensors that facilitate molecular detection.

**Fig. 1:**
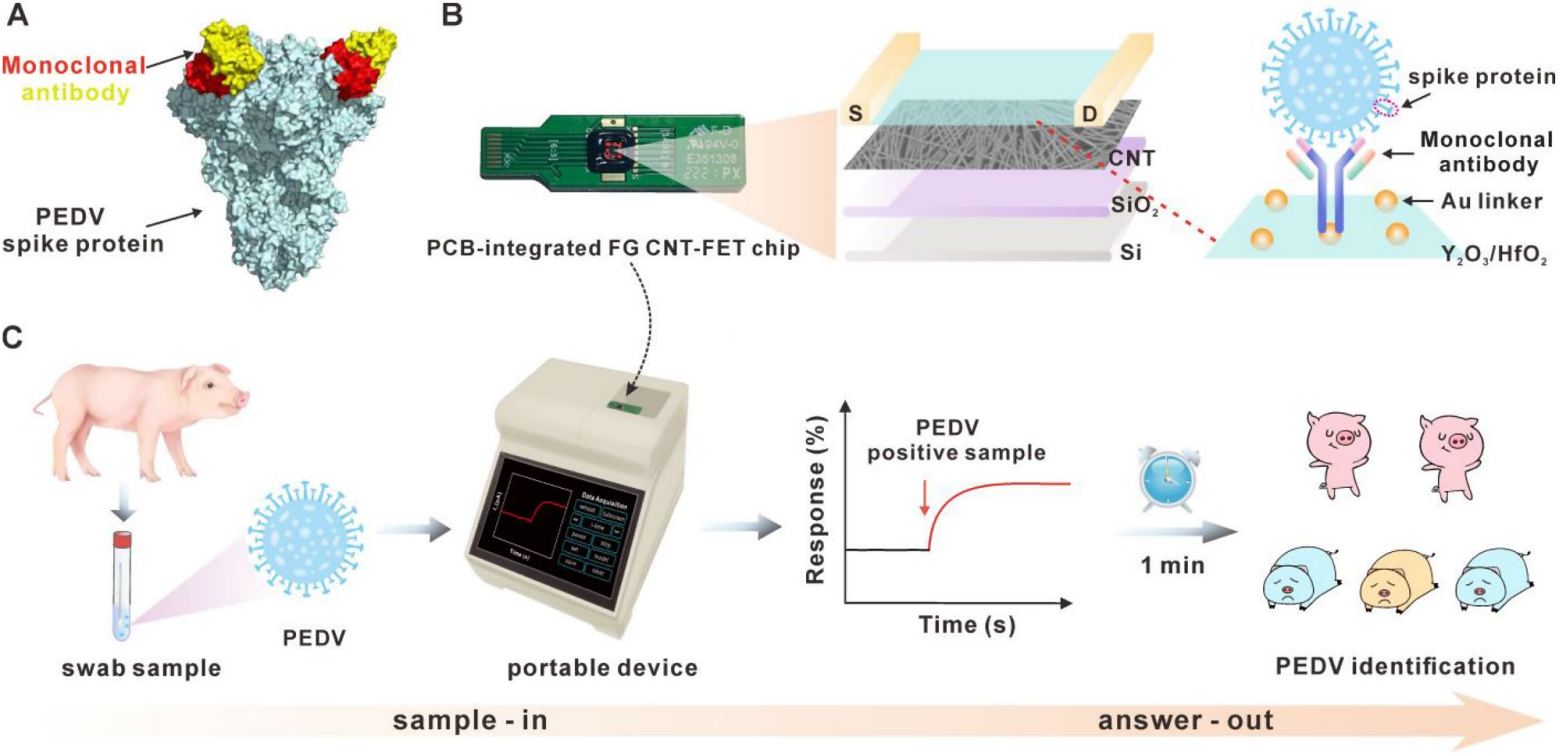
Schematic illustration of the FG CNT-FET biosensor for PEDV detection. (**A**) Structure of the complex formed by the monoclonal antibody and PEDV spike protein. (**B**) Diagram of the structure of the PCB-integrated FG CNT-FET chip. (**C**) Schematic diagram of the portable device for PEDV identification

In this study, we develop a FG CNT-FET biosensor functionalized by a mAb as a molecular recognition element for PEDV detection (Fig. 1). Firstly, we prepare and purify mAb that specifically binds with PEDV S-protein (Fig. 1A). The utilization of mAbs as molecular recognition elements for sensors offers distinct advantages over polyclonal antibodies, including ease of synthesis, high purity, cost-effectiveness, heightened specificity by targeting conserved viral regions, and a substantial reduction in false negatives. On the other hand, we fabricate a highly stable and homogeneous FG CNT-FET chip and customize an integrated portable device. In order to achieve better biofunctionalization and environmental stability, a high κ dielectric layer of 3 nm yttrium oxide (Y_2_O_3_) / 10 nm hafnium oxide (HfO_2_) is processed on the chip channel, followed by the deposition of Au nanoparticles (AuNPs) as a linker to immobilize mAbs (Fig. 1B). When the swab sample containing PEDV is incubated with the fabricated chip, the introduction of negative charge leads to an increase in hole carriers in the channel and a rise in current (I_ds_) between the drain and source. Finally, by inserting the chip into the portable device, the PEDV-induced electrical response can be recorded in real time, enabling rapid on-site detection of PEDV within 1 min (Fig. 1C). Remarkably, this diagnostic approach exhibits 100% consistency with quantitative reverse transcription polymerase chain reaction (qRT-PCR) results.

## Experimental methods

### Materials and chemical reagents

PEDV spike protein, monoclonal antibody, lilly laboratories cell-porcine kidney 1(LLC-PK1), african green monkey kidney (Vero-CCL81), swine testis (ST), meat animal research center-145 (Marc145), human rectal tumor 18 (HRT18) and human embryonic kidney (HEK293T) cells, PEDV, porcine deltacoronavirus (PDCoV), transmissible gastroenteritis coronavirus (TGEV), porcine reproductive and respiratory syndrome virus (PRRSV), porcine hemagglutinating encephalomyelitis virus (PHEV), anti-TGEV, anti-PDCoV, anti-SADS-CoV, anti-PRRSV and anti-PHEV were supplied by Prof. Wentao Li from Huazhong Agricultural University. Swine acute diarrhoea syndrome coronavirus (SADS-CoV) was kindly provided by Prof. Yongchang Cao from Sun Yat-sen University. Dulbecco’s modified eagle medium (DMEM), fluorescent secondary antibodies and protein A-sepharose^@^ 4B conjugate were provided by Life Technologies (California, USA). Fetal bovine serum (FBS), bovine serum albumin (BSA), glutaraldehyde and mercaptoethylamine were purchased from Sigma-Aldrich (St.Louis, USA). Alexa Fluor 488-conjugated AffiniPure Goat Anti-Human IgG (H + L) and polyclonal cat IgG (catalog no. 002-000-003) were provided by Jackson Immuno Research Laboratories Inc. (Pennsylvania, USA). Sterile phosphate buffer solution was obtained from Boster Biological Technology (Wuhan, China). Throughout the study, a Millipore water purification system (18.2 MΩ resistivity, Milli-Q Direct 8) was applied to provide ultrapure water.

### Preparation of mAb

The mAb targeting the PEDV G2b strain S1-protein (GDU, Genbank: KU985230.1) was produced according to the previously described method [43]. Briefly, RNA from subcloned hybridoma monoclonal cell lines was extracted and cDNA was obtained using Roche Reverse Transcription Kit. The heavy and light chains of the specific mAb were amplified [44] and cloned into the vector pFUSE2ss-CHIg-hG1 and pFUSE2ss-CLIg-hK, respectively. The recombinant plasmids pFUSE2ss- CHIg-PEDV-hG1 and pFUSE2ss-CLIg- PEDV-hK were co-transfected in HEK293T cells. The mAbs expressed in cell supernatants were collected and purified by protein A-sepharose^@^ 4B conjugate 5 days after transfection. The purified mAbs were further verified by sodium dodecyl sulfate‒polyacrylamide gel electrophoresis (SDS-PAGE) and stored at -80℃ for subsequent experiments.

### Indirect immunofluorescence assay

LLC-PK1, Vero-CCL81, ST, Marc145, and HRT18 cells were maintained in DMEM supplemented with 10% FBS, 2 mM L-glutamine, 100 U/mL penicillin, and 100 µg/mL streptomycin, and then seeded into 24-well plates, respectively. Confluent cells were infected with SADS-CoV, PEDV, PDCoV, TGEV, PRRSV and PHEV at a multiplicity of infection (MOI) of 0.1. SADS-CoV was propagated and titrated in LLC-PK1 cells. PEDV was propagated and titrated in Vero-CCL81 cells. TGEV and PDCoV were propagated and titrated in ST cells. PRRSV was propagated and titrated in Marc145 cells, and PHEV was propagated and titrated in HRT18 cells. After 24 h, the cells were fixed at room temperature for 15 min with 4% paraformaldehyde and then permeabilized for 15 min with TritonX-100 (1:1000). Subsequently, cells were blocked with 5% BSA in PBS for 1 h at room temperature. The indicated primary antibodies were added to the cells and incubated for 1 h at room temperature, followed by treatment with fluorescent secondary antibodies. The fluorescent signals were observed using fluorescence microscope (Olympus IX73, Japan) after 4’,6- diamidino-2- phenylindole (DAPI) staining.

### Bio-layer interferometry analysis

Binding of PEDV S-protein and PEDV S-specific mAbs was measured on the bio-layer interferometry platform using protein A sensors (FortéBio, USA) as described previously method [45]. Briefly, a biosensor was coated with S-Fc protein (1.6 µg/mL) until saturation, followed by blocking with polyclonal cat IgG for 200s. Subsequently, the sensor was exposed to PEDV S-specific mAb (1 mg/mL) for 400s, and the interferometry signal was recorded. Finally, the probe was immersed in the buffer and dissociated for 300 s.

### Fabrication of FG CNT-FET biosensors

The chip fabrication process built on findings from previous report [39]. Briefly, pure polymer-sorted carbon nanotube solutions were deposited onto Si/SiO_2_. Ti/Pd/Au (0.3 nm/40 nm/30 nm) electrodes were evapotrated by using electron beam evaporation (EBE) to act as the source and drain. Channel regions were formed by performing reactive ion etching. Y films were created via EBE, then heated in air at 270 °C for 30 min to produce yttrium oxide (Y_2_O_3_). Hafnium oxide (HfO_2_) was grown by atomic layer deposition. Au nanoparticles were deposited onto HfO_2_ via magnetron sputtering. Photoresist (S1813) was then used to passivate the contacts and wires of the FG CNT-FET, protecting them from the electrolyte.

### Functionalization of FG CNT-FET biosensors

10 µl of 10 mM mercaptoethylamine solution was added to the channel region of the FET sensor for 12 h at room temperature. The thioglycolic group formed Au-S bonds on the surface of AuNPs. A 5% glutaraldehyde solution was added and incubated for 2 h at room temperature. Next, 50 µg/mL mAb solution was added to the channel and conjugated with glutaraldehyde at 4 ℃ for 12 h. Unbound antibody molecules were removed by sequential rinsing with 1×PBS containing 0.2% SDS, 1×PBS, and ultrapure water. Finally, after being blocked with 1 mg/mL BSA for 1 h at room temperature to prevent the nonspecific bindings of the channel surface, washing with ultrapure water and blow-drying with nitrogen, the mAb-functionalized FG CNT-FET biosensor was obtained.

### Electrical measurement

For static testing, 10 µL of spike protein or PEDV was added to the mAb-functionalized biosensor and incubated for 10 min and then the transfer characteristic curve of the sensor was measured and recorded by a semiconductor characterization system (Keithley 4200-SCS, USA) connected to a probe station (Ever Being BD-6, China) at V_d_ = -0.2 V. The liquid-gate electrolyte was 0.001×PBS. For dynamic sensing, the drain current-time (I_ds_-t) curves at V_d_ = -0.2 V and V_g_ = -0.6 V were monitored. A portable FG CNT-FET biosensor device was further used to detect clinical samples.

### Preparation of clinical samples

A set of 40 oral and fecal swab clinical samples were kindly provided by Prof. Wentao Li from the Animal Disease Diagnostic Center, Huazhong Agricultural University. Among these samples, 20 were identified as positive samples and the other 20 were determined to be negative samples by qRT-PCR. The samples were placed in sample collection tubes containing 1 ml of PBS, mixed vigorously and allowed to stand for 3 min at room temperature, and the supernatant was analysed. And then, these processed samples were randomly numbered S1-S40 for double-blind test.

## Results and discussion

### Production, purification, and validation of mAb

**Fig. 2:**
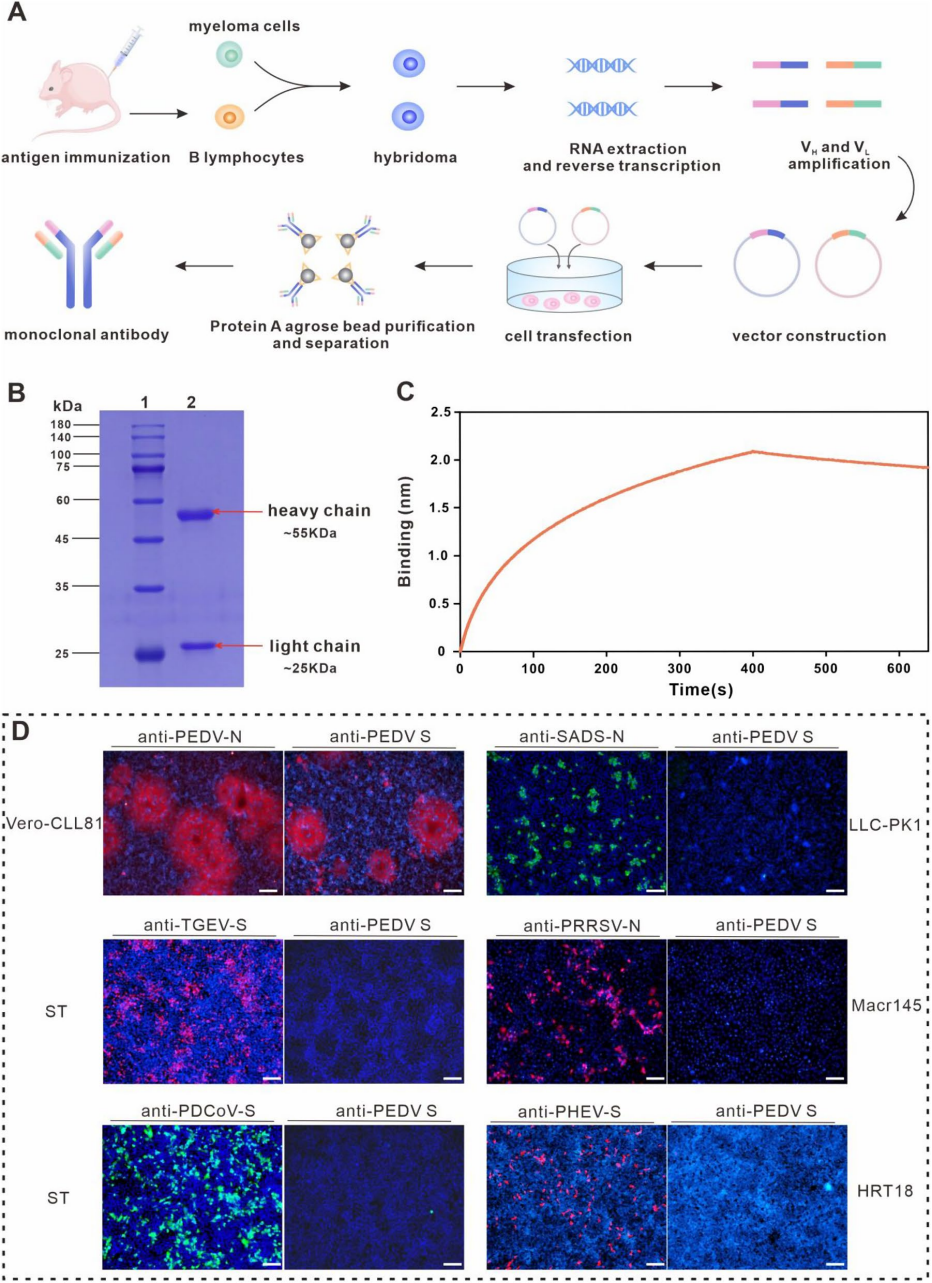
Production and performance validation of mAb. (**A**) Schematic diagram of the process of mAb production. (**B**) Validation of the mAb by SDS-PAGE. Lanes 1 and 2 indicate marker and mAb, respectively. SDS-PAGE displays the heavy (- 55 kDa) and light (- 25 kDa) chain bands of mAb. (**C**) Determination of mAb binding to S-protein by BLI. (**D**) Specific binding of mAb verified by IFA. Scale bar in the image represents 100 μm

The mAb targeting the PEDV variant strain S-protein was generated as described in Fig. 2A. The purified mAb was further verified by SDS-PAGE and bands were demonstrated to migrate at the anticipated molecular weights (55 kDa for heavy chain and 25 kDa for light chain) (Fig. 2B). To confirm the binding capacity of generated mAb to PEDV S-protein, we performed bio-layer interferometry (BLI) assay and calculated the dissociation constant (K_d_) by Fortebio Data Analysis 7.0 software. The K_d_ value was 4.84 nM. As depicted in Fig. 2C, significant binding signals could be observed, which showed the high affinity between the generated mAb and the PEDV S-protein. Furthermore, the specificity of mAb was characterized by indirect immunofluorescence assay (IFA) using antibodies specific for PEDV N-protein, SADS-CoV N-protein, TGEV S-protein, PRRSV N-protein, PDCoV S-protein and PHEV S-protein. As shown in Fig. 2D, SADS-CoV, PEDV, PDCoV, TGEV, PRRSV and PHEV all had successful infection, yet PEDV-specific immunofluorescence signal was only detected in PEDV-infected Vero-CCL81 cells. The results indicate that the prepared mAb possesses decent specificity. Notably, the mAb was prepared using a highly pathogenic PEDV strain that reacts with both the currently prevalent strains, but not with the less pathogenic group of viruses, which is of great importance in the fight against the current PEDV pandemic.

### Fabrication and characterization of FG CNT-FET chip

**Fig. 3:**
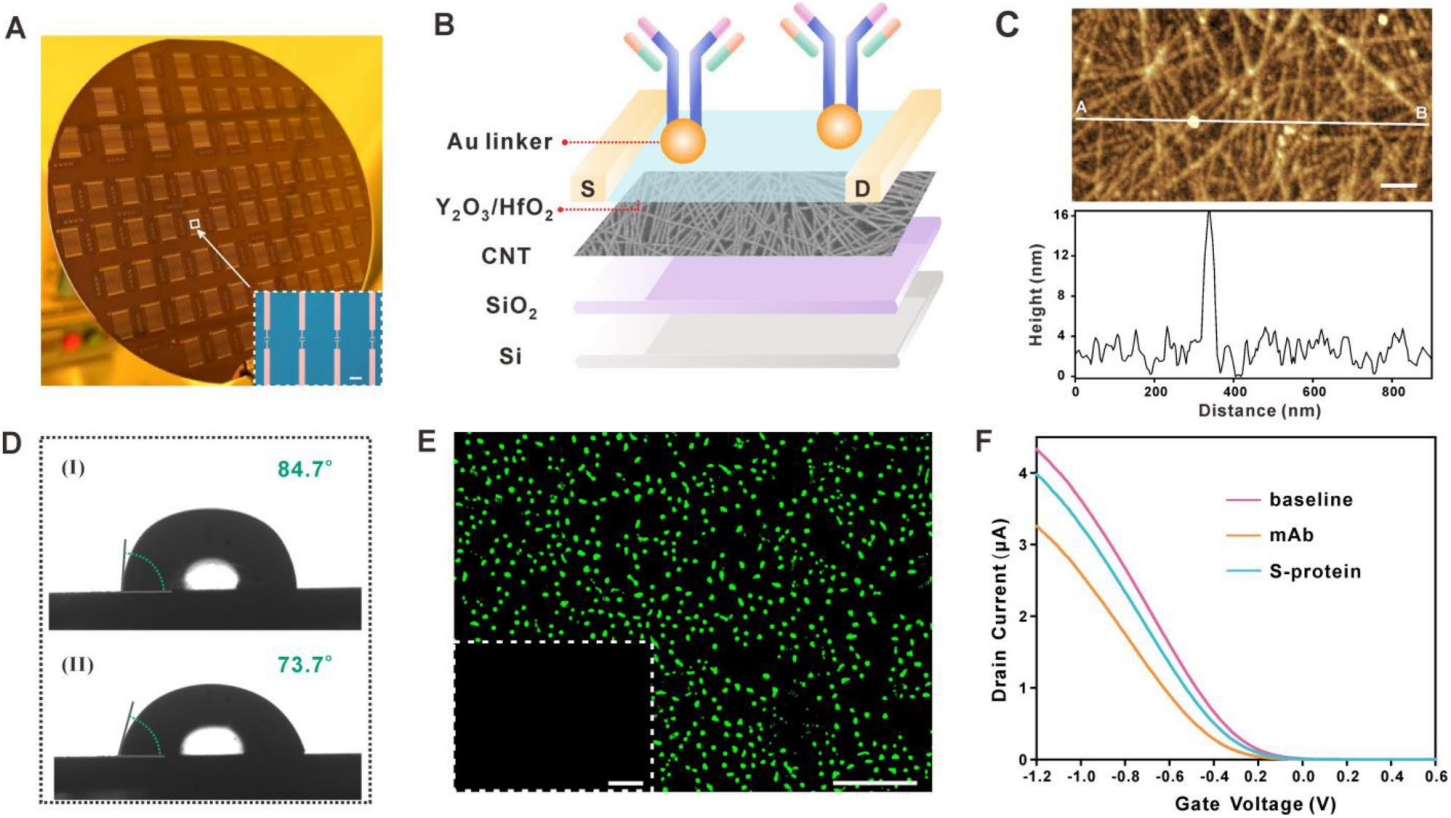
Characterization of FG CNT-FET sensor. (**A**) Photo image of the FG CNT-FET sensor array on a 4-inch silicon wafer. The inset shows optical image of four FG CNT-FET sensors (scale bar, 200 μm). (**B**) Geometric diagram of the FG CNT-FET sensor. (**C**) AFM images and height profiles of antibody functionalized FET chip (scale bar, 100 nm). (**D**) Contact angle of the FET chip surface before (**I**) and after (**II**) mAb functionalization. (**E**) Fluorescence microscopy image of mAb-functionalized FET chip incubated with an Alexa 488-conjugated fluorescent secondary antibody (scale bar, 20 μm). The inset shows fluorescence microscopy image of the FET chip without mAb functionalization (scale bar, 20 μm). (**F**) Transfer characteristic curve of the FG CNT-FET sensor in the mAb functionalization and detection of S-protein processes

The FG CNT-FET chips were manufactured as previously described [39]. Images of the FG CNT-FET chip structure are shown in Fig. 3A. It was composed of polymer-sorted CNTs with semiconducting purity higher than 99.99% as the channel material, Y_2_O_3_/HfO_2_ as the insulating layer, and AuNPs as the linker to immobilize mAbs (Fig. 3B). The scanning electron microscopy (SEM) in Figure S1A and S1B demonstrated that AuNPs were uniformly distributed on the Y_2_O_3_/HfO_2_ layer. Similar results of SEM could be observed by atomic force microscopy (AFM) in Figure S1C, indicating that the AuNPs were successfully assembled onto the chip surface. In order to characterise the successful functionalization of the FET chip with mAb, we first employed AFM to verify the biological coupling. Figure 3C shows an increase in signal intensity after mAb modification on the channel surface. Meanwhile, a contact angle test was also performed. As can be seen from the contact angle image in Fig. 3D, the contact angle of the chip decreased from 84.7° to 73.7° after mAb modification, which is caused by the fact that the hydrophilic group of antibody improved the hydrophilicity of the insulating layer surface. To further confirm that the mAb was ideally immobilized, Alexa Fluor 488-conjugated AffiniPure Goat Anti-Human IgG (H + L) was utilized to incubate with mAb-modified chip. Green fluorescence signal was observed only in the mAb immobilized chips as expected (Fig. 3E). Simultaneously, the drain current-gate voltage (I_d_-V_g_) transfer characteristic curves in Fig. 3F verified the FG CNT-FET sensor’s ability to detect the S-protein. Specifically, the sensor conducts electrons mainly through hole carriers. When a positively charged mAb (pI = 8.0) is introduced to the sensing interface of the sensor, an electrostatic gating effect is generated. This effect is then transferred to the CNT via gate dielectrics, causing a decrease in the hole carriers inside the CNT. Consequently, there is a reduction in the source and drain currents (I_ds_) of the sensor. Due to the addition of negatively charged S protein (pI = 5.85), the I_ds_ increases as a result of the increase in hole carriers inside the CNT.

### Stability, repeatability and regeneration

For rapid and POC assays, antibody-based sensors are typically recommended to be stored at 4 °C [46, 47] and used within a certain time after unpacking. To further evaluate the stability of the mAb-functionalized sensor under environmental condition of 25 °C and 50% relative humidity, 5 test groups were designed to detect the same concentration of the target. As displayed in Figure S2A, the signal response of the mAb-functionalized sensor to the S-protein remained constant within a specific range after 1, 2, 3, 4, and 5 h of exposure to ambient conditions. The result illustrates that the sensors still possess excellent stability under environmental conditions, which is benefit from outstanding protection of the sensing channel by the Y_2_O_3_/HfO_2_ dielectric layer.

To investigate the repeatability of the sensors, 8 sensors were used to detect 11.4 pg/mL of S-protein in order to assess the precision. As shown in Figure S2B, the signal response of 8 sensors remained stable with an RSD of 3.26%, which indicates that our sensor has a superior uniformity of response to the target and provides a robust device platform with potential for PEDV detection.

Excellent regeneration of biosensors is one of the most critical factors to reduce their detection cost in practical applications. Hence, the regeneration of FET biosensors was investigated. First, S-protein (11.4 pg/mL) was added to the surface of the mAb-functionalized chip for detection. Subsequently, S-protein was dissociated from the chip by previously reported method [48–50] i.e., 1 M NaCl was used to disrupt the binding between S-protein and mAb. Then, such processes of dissociation and immunoconjugation were repeated 3 times and the corresponding transfer characteristic curves were recorded. It was found that the efficiency of the secondary and thirdly immune binding relative to the primary binding was 95.69 and 89.05%, respectively (Figure S2C), indicating that the prepared biosensor possessed excellent regenerative properties. This reveals that the chip is expected to be re-used in future applications to reduce the detection costs.

### Sensitivity of FG CNT-FET biosensor for PEDV S-protein detection

To systematically assess the detection performance of our developed FG CNT-FET sensors, a series of PBS solutions containing different concentrations of recombinant S-protein were used for static measurement. First, the transfer characteristic curves were recorded before and after the addition of S-protein. As shown in Figure S3A, due to the introduction of negatively charged S-protein, as expected, the transfer curves raised and the I_ds_ increased with its concentration ranging from 11.4 fg/mL to 1.14 ng/mL. We quantified the response of the sensor to the target, which is defined as the relative change in drain current at a gate voltage of -1.2 V (response = ΔI_ds_ /I_ds0_, ΔI_ds_ = I_ds_ -I_ds0_, where I_ds0_ and I_ds_ represent the drain current value before and after target incubation, respectively). As demonstrated in Figure S3B, the calibration plots indicated a good linear relationship (R^2^ = 0.9957) between the response and the logarithmic concentrations. Based on the linear regression equation and the 3-fold signal-to-noise ratio, the limit of detection (LoD) was estimated to be 8.1 fg/mL.

### Analytical performance of PEDV by FG CNT-FET biosensor

**Fig. 4:**
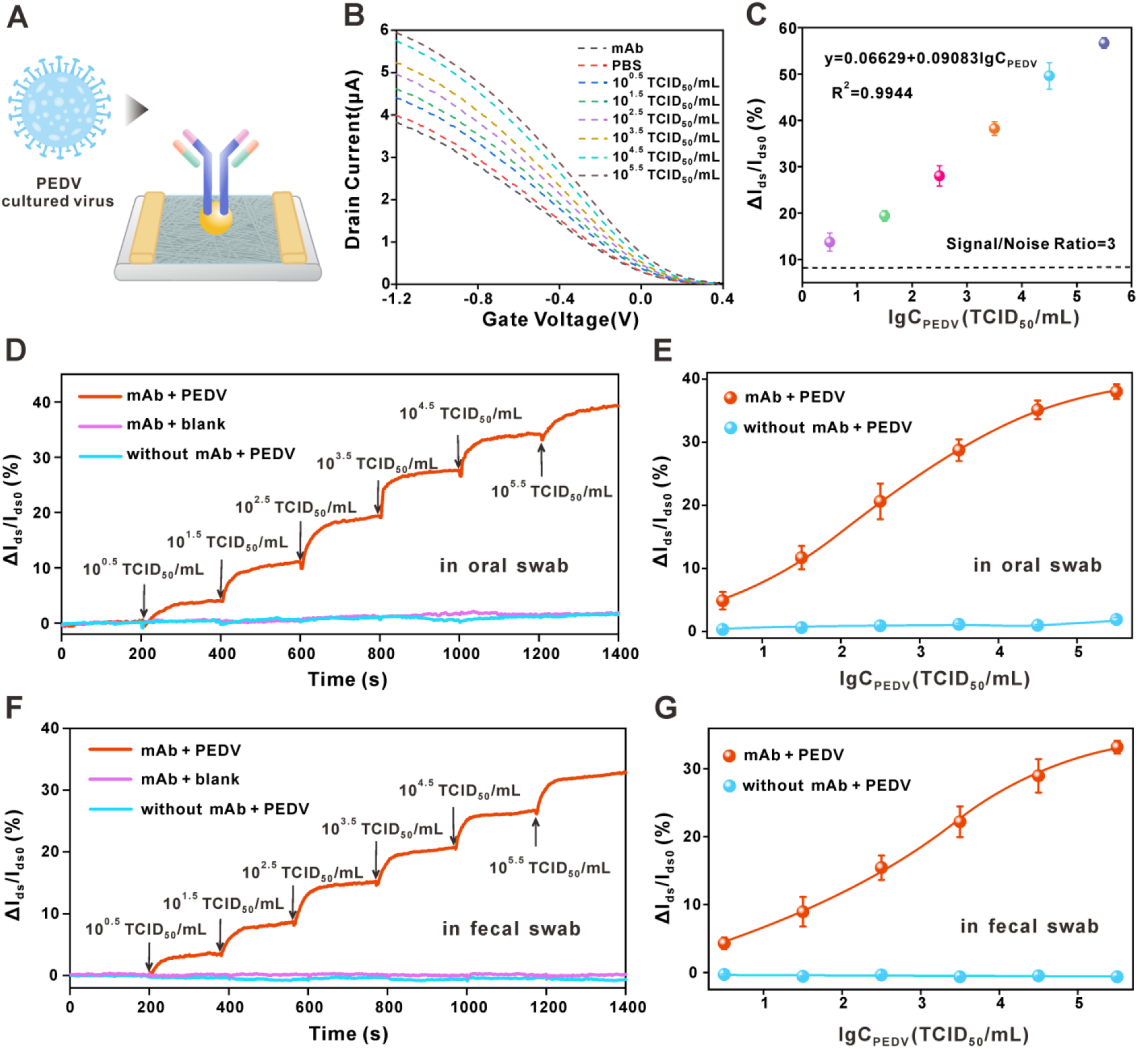
Detection of PEDV by FG CNT-FET biosensor. (**A**)Schematic diagram for the FG CNT-FET sensor for detection of cultured PEDV. (**B**) The change in the transfer curve of the FG CNT-FET biosensor was recorded after the introduction of different PEDV concentration ranging from 10^0.5^ TCID_50_/mL to 10^5.5^ TCID_50_/mL. (**C**) The response (ΔI_ds_/I_ds0_) of sensor as a function of the logarithm of PEDV concentration (*n* = 3). (**D**) Real-time responses of FG CNT-FET sensor to different concentrations of PEDV in oral swab and (**E**) related dose-dependent response curves (*n* = 3). (**F**) Real-time responses of FG CNT-FET sensor to different concentrations of PEDV in fecal swabs and (**G**) related dose-dependent response curves (*n* = 3). Error bars are determined by the standard deviation of three measurements

Next, we evaluated the ability of the FG CNT-FET biosensor to detect PEDV in culture media. To this end, we cultured PEDV in Vero cells and diluted to serial concentrations, then applied the biosensor for analysis (Fig. 4A). As illustrated in Fig. 4B, the I_ds_ of sensor was positively correlated with the virus concentration, which is attributed to the introduction of negatively charged virus into the sensing interface, allowing for an increase in hole carriers between the source and drain. The current response curves were linear from 10^0.5^ to 10^5.5^ TCID_50_ /mL (Fig. 4C). Based on 3-fold signal-to-noise ratio, the LoD of the FG CNT-FET biosensor was 10^0.14^ TCID_50_ /mL. Compared to other PEDV sensing platforms, the FG CNT-FET biosensor exhibits excellent detection sensitivity (Table S1). This superior performance may benefit from the following points, namely (i) the introduction of the high κ dielectric layer of Y_2_O_3_/HfO_2_ avoids the complex effects of multiple mechanisms on channel sensing in the test solution environment; (ii) strong affinity between mAb and PEDV S-protein. The high sensitivity may reduce the false negative rate in early screening for PEDV infection. In addition, compared to qRT-PCR, the FG CNT-FET biosensor eliminates the need for signal amplification strategies and lysis processing, enabling on situ detection of PEDV.

The diameter of a single PEDV particle is approximately 100 nm, whereas the Debye length (λ_D_) of the physiological solution is less than 1 nm [51]. Hideshima et al. demonstrated that the charges of the viral particles that exist near the interface could be successfully detected by the FET biosensor under nearly physiological conditions. The viral particles may exclude the solution present between the viral surface and the sensor surface, reducing the effectiveness of the Debye screening [52]. Park et al. concluded that the large size of the virus particles allowed more charges to interact with the electronic channels of the sensor, eliciting a greater sensing response [53]. These results suggest that FET biosensor can overcome the λ_D_ limitation to detect virus particles. In addition, we used a low ionic strength solution (0.001 × PBS, λ_D_ ≈ 22 nm) as the detection electrolyte, resulting in a high response signal. However, it is worth of mentioning that the diluted buffer solution may decrease the specific binding efficiency between the antibody and the target [54].

High specificity is the key to improve the diagnostic accuracy and reduce misdiagnosis. Subsequently, the specificity of FG CNT-FET sensor was investigated. TGEV, PDCoV, SADS-CoV, PRRSV and PHEV were selected to use as interfering viruses. This is due to the fact that these viruses are all porcine coronaviruses, and the symptoms caused by their infections highly overlap with those of PEDV infections. As displayed in Figure S4A, the response to PEDV was remarkably higher than those of 5 interfering viruses and was statistically different. In addition, the results of FG CNT-FET sensors functionalized with non-specific antibodies (anti-TGEV, anti-PDCoV, anti-SADS-CoV, anti-PRRSV and anti-PHEV) for PEDV detection were demonstrated in Figure S4B, and it can be observed that only the PEDV-mAb-functionalized device produced a significantly high signal response to PEDV. Consequently, the developed biosensor exhibits outstanding specificity, which provides an opportune future for PEDV detection.

### Detection of PEDV in oral and fecal swabs by FG CNT-FET biosensor

Biological samples contain complex matrix environments and interfering components. To ensure practical relevance, the immunity of our biosensors to biological samples was first confirmed prior to testing actual samples. Briefly, PEDV was added to oral and fecal swab samples from healthy pigs, respectively. As displayed in Figure S5A, S5B, the transfer characteristic curve positively shifted with increasing PEDV concentrations and achieved relatively low background. Strikingly, the response of the sensor was still large even with a 10^0.5^ TCID_50_/mL of PEDV. The results demonstrate that the sensor possessed a remarkably high sensitivity, even in complex matrix environments. To evaluate whether the sensor can be used for rapid diagnostics, dynamic tests were further performed in negative oral swab and fecal swab samples. The response of the bare FG CNT-FET sensor to PEDV and the mAb-modified sensor to healthy samples without the target were conducted as control experiments. As shown in Fig. 4D, 4F and S6, the introduction of PEDV induced a sharp increase in current response and approached equilibrium within 1 min (red line), and the change amplitude increased with increasing concentration, which is consistent with the trend in static test. However, in the presence of PEDV, the chip of unfunctionalized mAb showed a negligible response change (blue line). Another control group with an equal volume of PBS instead of PEDV also showed a similar result (purple line), indicating that the response of the chip is due to the antigen-antibody binding rather than the disturbance of the solution volume change. In addition, Fig. 4E and 4G displayed the linear response of the sensor with increasing target at all studied concentrations in oral swab and fecal swab samples from healthy pigs. These results demonstrate that the sensors can rapidly detect PEDV at a lower concentration, which may exhibit great potential in the rapid detection field.

### Initial validation of integrated portable FG CNT-FET sensing platform for point-of-care testing of PEDV

To achieve the goal of offering efficient point-of-care testing for farms, a sensing platform consisting of a portable sensing device and an integrated FG CNT-FET chip provided by Peking University was used to detect PEDV [55]. As displayed in Figure S7A and S7B, the portable device could be used directly for data acquisition and analysis. The FG CNT-FET sensors were packaged as plug-in chips in a mass-production way (Figure S7C). We first investigated the analytical performance of the sensing platform for the farm samples. Generally, sample dilution to minimize non-specific binding and reduce viscosity is necessary for rapid antigen detection [56]. To this end, we first measured the response of samples with different dilution ratios in order to evaluate the sensor’s accurate sensing capability. As shown in Figure S8, even with 10^4^ dilutions, the current response still reached 2.83%, indicating the high sensitivity of the sensor. It is worth noticing that the responses derived from the 10^5^ dilution were less than 2 times the signal of the control sample and therefore could not be quantified. Subsequently, the ability of the sensing platform to distinguish between PEDV-positive and negative oral swab samples was investigated. 3 PEDV-positive and 3 negative oral swab samples validated by qRT-PCR were utilized for testing. qRT-PCR’s amplification cycle threshold (Ct) cut-off value of 32 was determined to distinguish PEDV positive and negative subjects. The ΔI_ds_/I_ds0_*versus* time curves in Figure S9A displayed that the positive samples generated a dramatic signal response within seconds and plateaued at 1 min after injection, demonstrating that PEDV in oral swab samples could be detected by our platform within 1 min. Notably, our platform has a much lower response time than LFA and ELISA and can be implemented without the need for specialized laboratories and skilled personnel, which is more suitable for on-site testing. Figure S9B indicated that the response value of the sensor was approximately 10 times higher for positive samples than for negative samples. These results initially confirm the potential of the sensing platform we developed for rapid detection of PEDV. However, the effect of excessively small sample size and subjective bias of the experimenter on the results cannot be ignored, so further experimental protocols need to be designed to verify the performance of sensing platform.

### Double-blind testing of PEDV detection by the integrated portable FG CNT-FET sensing platform

**Fig. 5:**
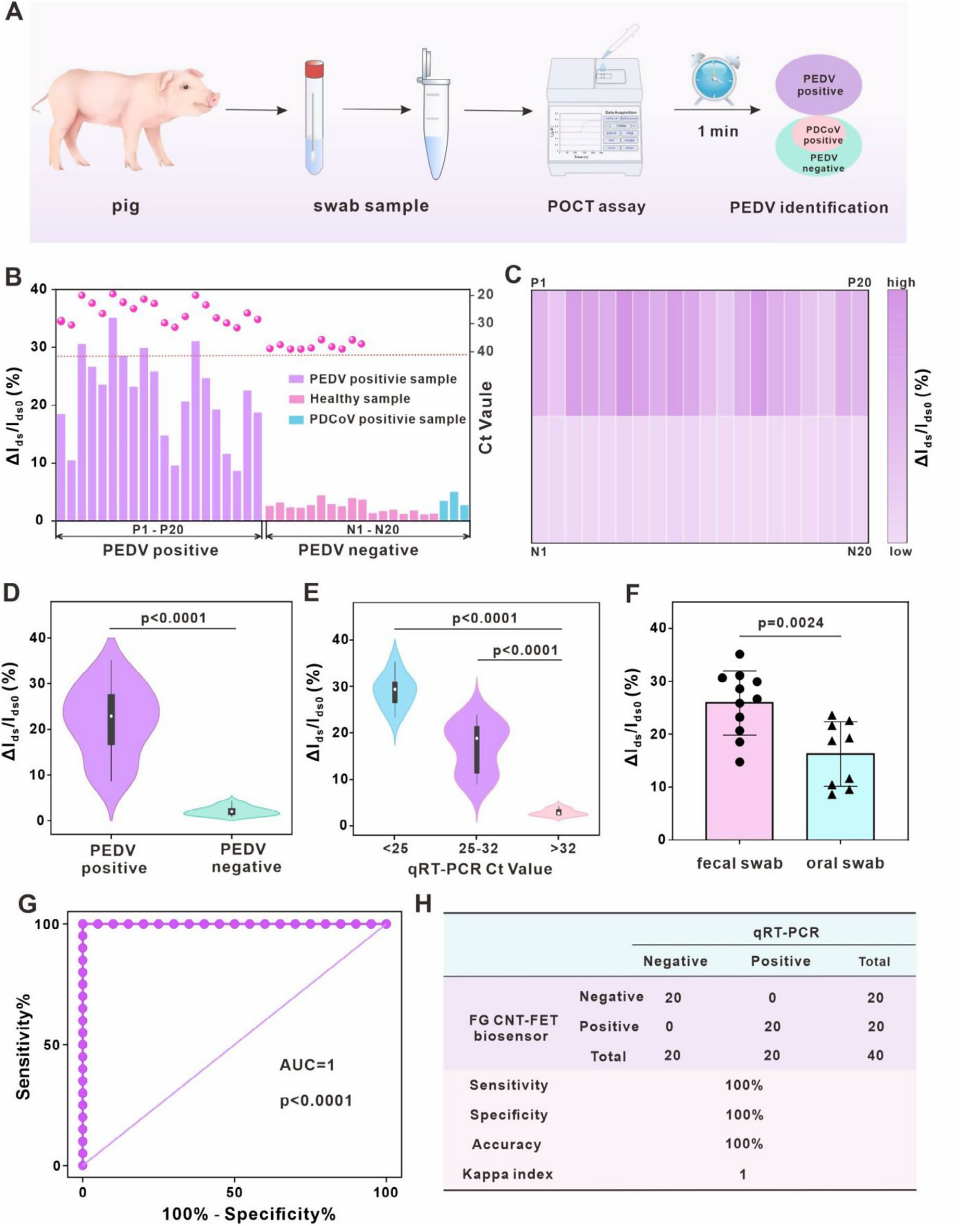
Double-blind testing of PEDV detection by an integrated portable FG CNT-FET sensing platform. (**A**) Schematic diagram of the integrated portable device for the PEDV detection of swab samples. (**B**) Response of sensing platform to PEDV-positive (P1-P20) and PEDV-negative (N1-N20) swab samples. (**C**) Heatmap showing the viral load levels of 40 swab samples with sensing platform. (**D**) T-test of the signal response of the two groups (positive and negative). (**E**) T-test of the signal response of samples with different Ct values. (**F**) T-test of the signal response of oral swab and fecal swab samples. (**G**) ROC analysis of positive and negative samples based on the integrated FG CNT-FET sensing platform. (**H**) Kappa analysis for diagnostic results of the integrated FG CNT-FET sensing platform and qRT-PCR

After a reliable and convincing preliminary feasibility study for laboratory validation of the sensing platform for PEDV detection in spiked and small numbers of samples, a double-blind testing of PEDV in a cohort confirmed by qRT-PCR was performed to further verify efficacy. Owing to the similarity of clinical symptoms, it remained challenging to discriminate effectively between PEDV and PDCoV, which points to the importance of differential diagnosis. We analyzed 40 anonymous samples from farms, of which 20 were negative samples (containing 3 PDCoV positive samples) and 20 were positive samples. The cut-off value for determining positive and negative by qRT-PCR was 32. The schematic of our sensing platform for PEDV detection in swab sample was described in Fig. 5A. Additionally, Table S2 summarized in detail the Ct values of the samples and results of double-blind testing. The signal responses of 40 samples were exhibited and the results are shown in Fig. 5B and C. The signal response in PEDV positive samples was more intense compared to health samples and PDCoV positive samples, signifying a clear differentiation. By the t-test results shown in Fig. 5D, the signal responses obtained from PEDV positive samples were much higher than those from PEDV negative samples with a statistically significant difference (*p* < 0.0001). Moreover, the relationship between sensing signals and the Ct values of qRT-PCR was compared. The sensor response was higher for samples with Ct values below 25 and decreased sequentially with increasing Ct value (Fig. 5E). Simultaneously, positive fecal swab samples yielded a stronger signal response than oral swab samples, implying a higher viral load (Fig. 5F). The result is in line with the reported lower levels of viral nucleic acids in oral swabs than in fecal swabs [57]. Subsequently, to assess the clinical performance of the trial, the receiver operating characteristic curve (ROC) was implemented. A striking accuracy was revealed in Fig. 5G, as we can observe, where the area under the curve (AUC) is 1. The optimal cut-off value (ΔI_ds_/I_ds0_ = 6.028%) is obtained from the point closest to the upper left corner of the ROC curve. The cut-off value was used to distinguish between YES/NO responses in PEDV-positive and negative samples. It is worth mentioning that this cut-off value is not immutable and it may vary with sample size [58]. On the basis of these results, Kappa analysis was utilized to assess the concordance between our sensor platform and qRT-PCR. Fig. 5H explicitly depicted a kappa index of 1, which indicates excellent consistency. Thus, it can be concluded that our sensing platform possessed a remarkable credibility in the direct detection of clinical samples, displaying a promising propensity to contribute to defeat PEDV.

## Conclusion

In summary, we have successfully prepared and purified mAbs that are capable of specifically binding with PEDV S-protein. Afterwards, the antibodies are further functionalized with FG CNT-FET chip to develop the immunosensor for the testing of PEDV particle. Contributing to high affinity for antigen-antibody and dielectric layer process of FG CNT-FET, our biosensor not only manifests promising stability and repeatability, but also empower the LoD down to 8.1 fg/mL (S-protein) and 10^0.14^ TCID_50_/mL (PEDV), which is the lowest value reported so far for PEDV antigen sensing. Moreover, a portable FG CNT-FET platform as a field-deployable chip readout achieves rapid and direct detection of PEDV within 1 min via a “sample-in-answer-out” approach. Validated in clinical cohorts, our sensing platform not only accurately distinguishes between PEDV-positive and PEDV-negative samples, but also allows for the identification of PEDV and PDCoV with highly consistent PCR results. Generally, the portable sensing platform with ultra-sensitivity, rapidness, on-site and user-friendly test format achieves point-of-care detection of PEDV on farms. In addition, by varying the antibodies used to construct the sensor, the portable sensing device can be utilized as a universal detection platform for other virus targets, which will be a powerful tool assisting epidemic disease management.

### Electronic supplementary material

Below is the link to the electronic supplementary material.

Supplementary Material 1

## Data Availability

No datasets were generated or analysed during the current study.
