# Supplementary material for: A portable transistor immunosensor for fast identification of porcine epidemic diarrhea virus
Source: J Nanobiotechnology. 2024 May 12;22:239. doi: 10.1186/s12951-024-02440-5 (PMC11089749; doi:10.1186/s12951-024-02440-5)
Supplement: Supplementary file 1 — Supplementary Material 1 [file 12951_2024_2440_MOESM1_ESM.doc]

**Supporting Information**

**A Portable Transistor Immunosensor for Fast Identification of Porcine Epidemic Diarrhea Virus**

Xiao Hu^1,2†^, Mengjia Zhang^3,4†^, Yiwei Liu^5^, Yu-Tao Li^1,7^, Wentao Li^3,4^, Tingxian Li^6^, Jiahao Li^1^, Xueqian Xiao^1^, Qigai He^3,4*^, Zhi-Yong Zhang^6*^, Guo-Jun Zhang^1,7*^

^1^School of Laboratory Medicine, Hubei University of Chinese Medicine, 16 Huangjia Lake West Road, Wuhan 430065, P.R. China

^2^Department of Pharmacy, Renmin Hospital, Hubei University of Medicine, Shiyan 442000, Hubei, P.R. China

^3^Agricultural Microbiology, College of Animal Sciences and Veterinary Medicine, Huazhong Agricultural University, Wuhan 430070, P. R. China

^4^The Cooperative Innovation Center for Sustainable Pig Production, Wuhan 430070, P. R. China

^5^Hunan Institute of Advanced Sensing and Information Technology, Xiangtan University, Hunan 411105, P. R. China

^6^Key Laboratory for the Physics and Chemistry of Nanodevices and Center for Carbon-based Electronics, School of Electronics, Peking University, Beijing 100871, P. R. China

^7^Hubei Shizhen Laboratory, Wuhan 430065, Hubei, P.R. China

* Corresponding author.

E-mail addresses: [zhanggj@hbtcm.edu.cn](mailto:zhanggj@hbtcm.edu.cn) (G.-J.Z)

^†^ These authors contributed equally to the work.

**Table of Contents：**

1. Figure S1. Characterization of FG CNT-FET sensor.
2. Figure S2. Stability, repeatability and regeneration of FG CNT-FET sensor.
3. Figure S3. Sensitivity of FG CNT-FET biosensor for S-protein detection.
4. Figure S4. Specificity of FG CNT-FET biosensor for PEDV detection.
5. Figure S5. Transfer characteristic curves of sensors in swab sample for the detection of different concentrations of PEDV.
6. Figure S6. I_d_-t curves of the FG CNT-FET sensor to different concentrations of PEDV in oral swab and fecal swab.
7. Figure S7. Photographs of the integrated portable device.
8. Figure S8. Signal response of portable devices to positive samples at different dilution factor.
9. Figure S9. Initial validation of portable devices detecting positive and negative samples.
10. Table S1. Comparison of the analytical performances of different methods for PEDV detection.
11. Table S2. Samples Ct values and double-blind testing results.

**
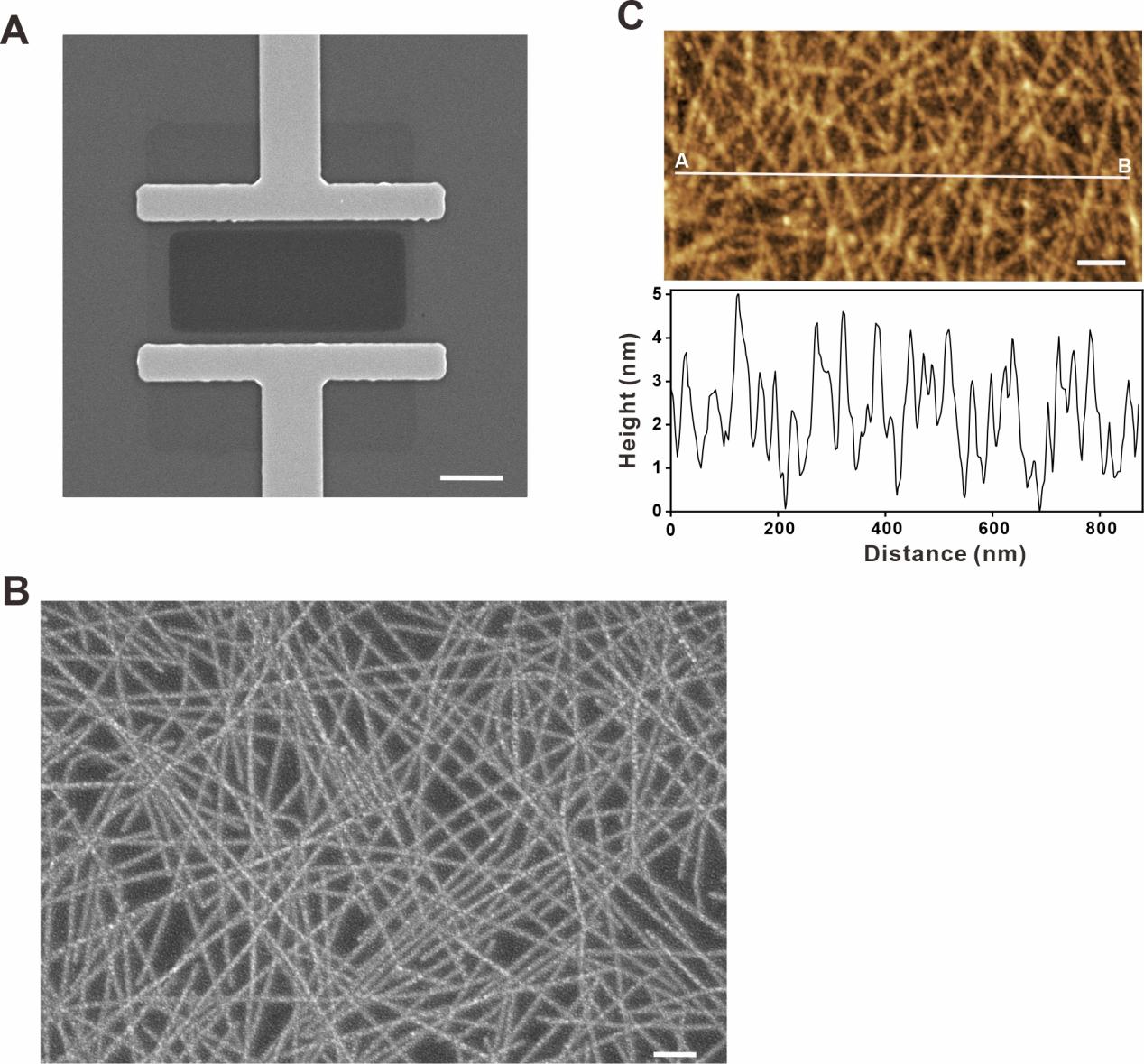
**

**Figure S1.** Characterization of FG CNT-FET sensor. (A) SEM image of a single FET sensor (scale bar, 10 μm). (B) SEM image displaying that AuNPs were uniformly distributed on the Y_2_O_3_/HfO_2_/CNT film in part of the channel region (scale bar, 100 nm). (C) AFM image and height profiles of without mAb functionalized FET chip (scale bar, 100 nm).


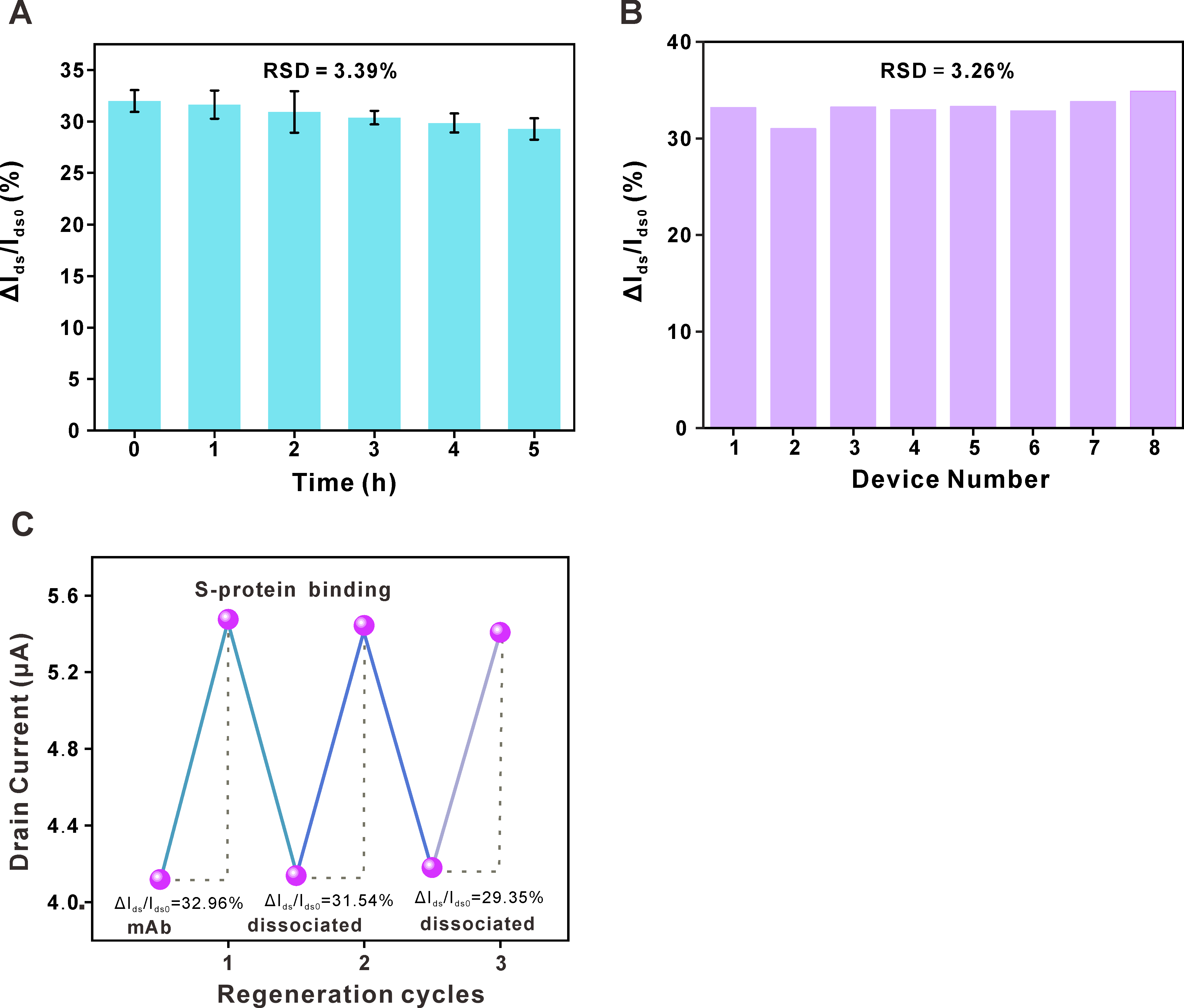


**Figure S2.** Stability, repeatability and regeneration of FG CNT-FET sensor. (A) Stability of mAb-functionalized FG CNT-FET chips stored under ambient conditions for 5 h. (B) Repeatability of 8 different FETs testing 11.4 pg/mL S-protein (n = 3). (C) Regeneration of the FG CNT-FET sensor for PEDV S-protein detection. The concentrations of S-protein and NaCl were 11.4 pg/mL and 1 M. Error bars are determined by the standard deviation of three measurements.

**
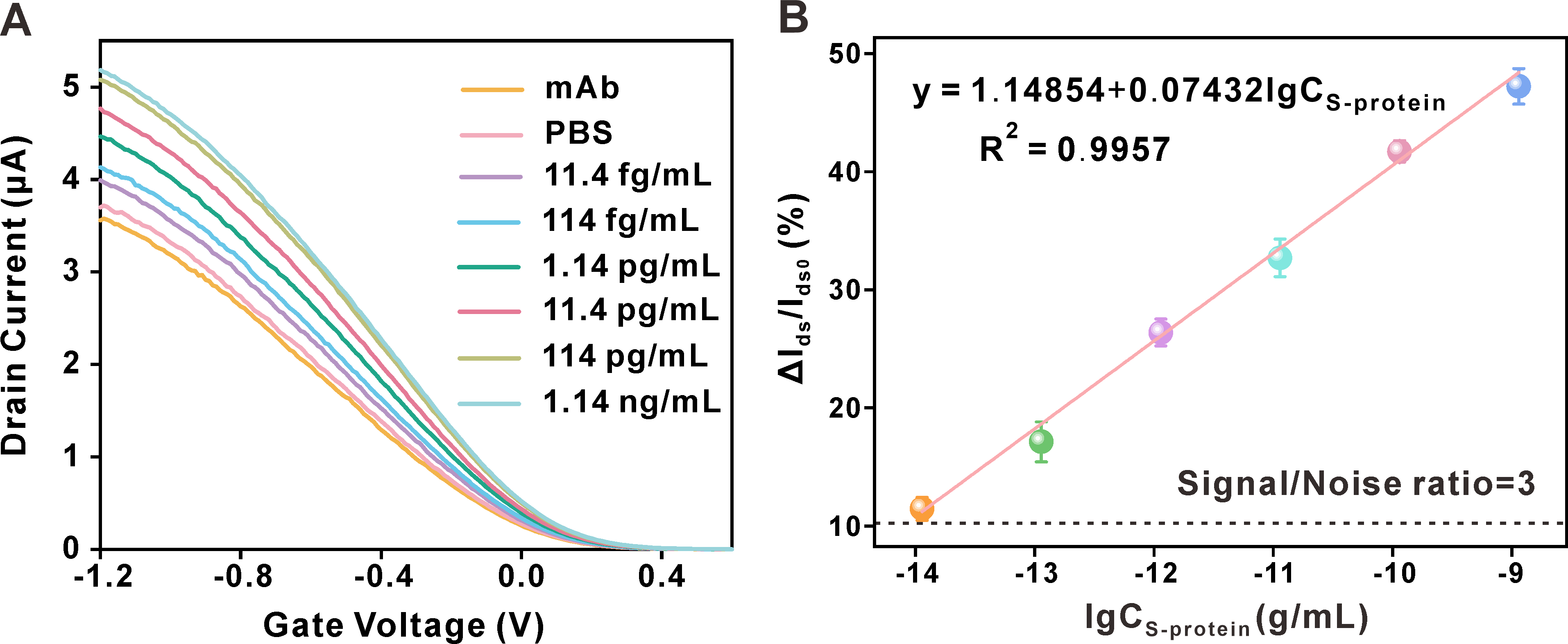
**

**Figure S3.** Sensitivity of FG CNT-FET biosensor for S-protein detection. (A) The change in the transfer curve of the FG CNT-FET biosensor was recorded after the introduction of different S-protein concentration ranging from 11.4 fg/mL to 1.14 ng/mL. (B) The response (ΔI_ds_/I_ds0_) of sensor as a function of the logarithm of S-protein concentration (n = 3). Error bars are determined by the standard deviation of three measurements.


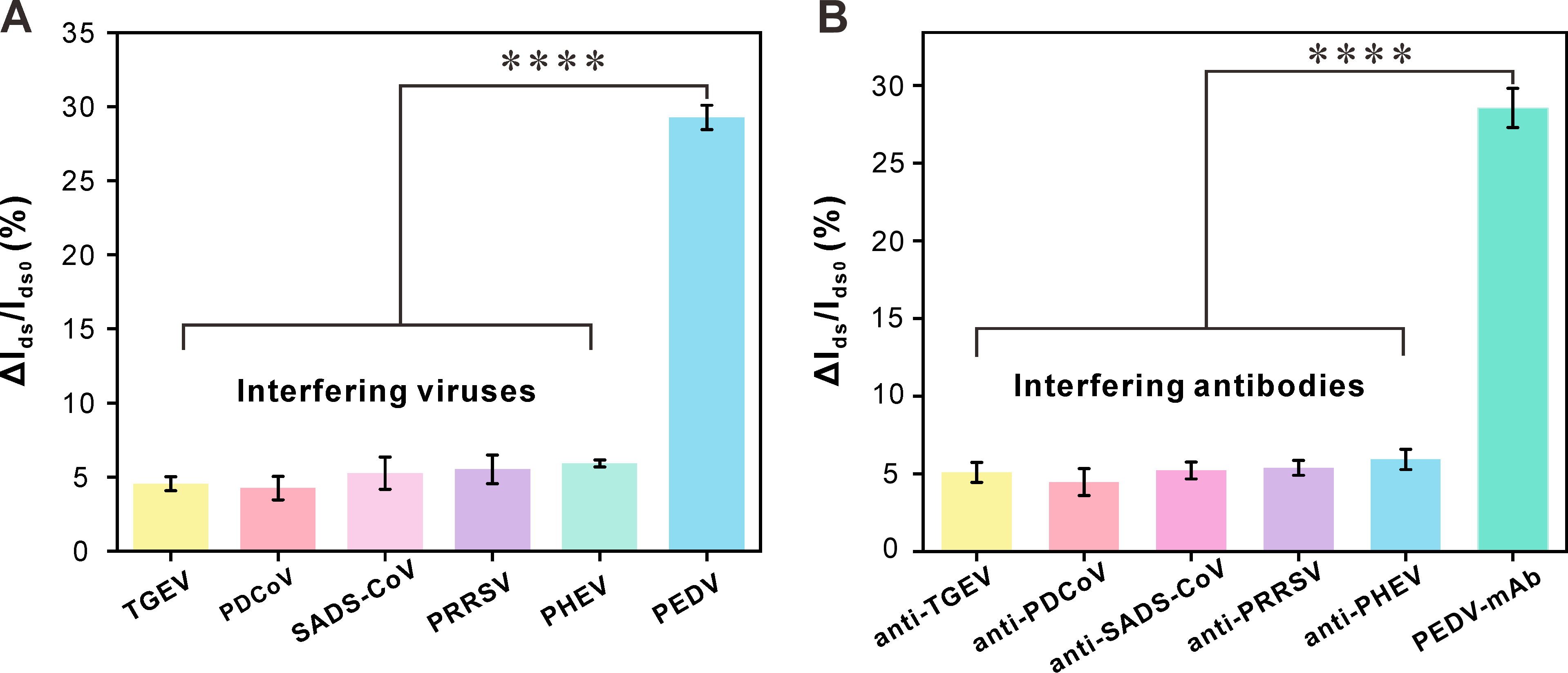


**Figure S4.** Specificity of FG CNT-FET biosensor for PEDV detection. (A) Specificity of mAb- functionalized FG CNT-FET sensors for PEDV. The sensors were incubated with 10^2.5^ TCID_50_/mL PEDV and 10^3.5^ TCID_50_/mL interfering viruses (TGEV, PDCoV, SADS-CoV, PRRSV and PHEV), respectively. (B) Specificity of anti-TGEV, anti-PDCoV, anti-SADS-CoV, anti-PRRSV, anti-PHEV and PEDV-mAb- functionalized sensors for PEDV (n = 3). Error bars are determined from the standard deviation of three measurements. **** p< 0.0001.


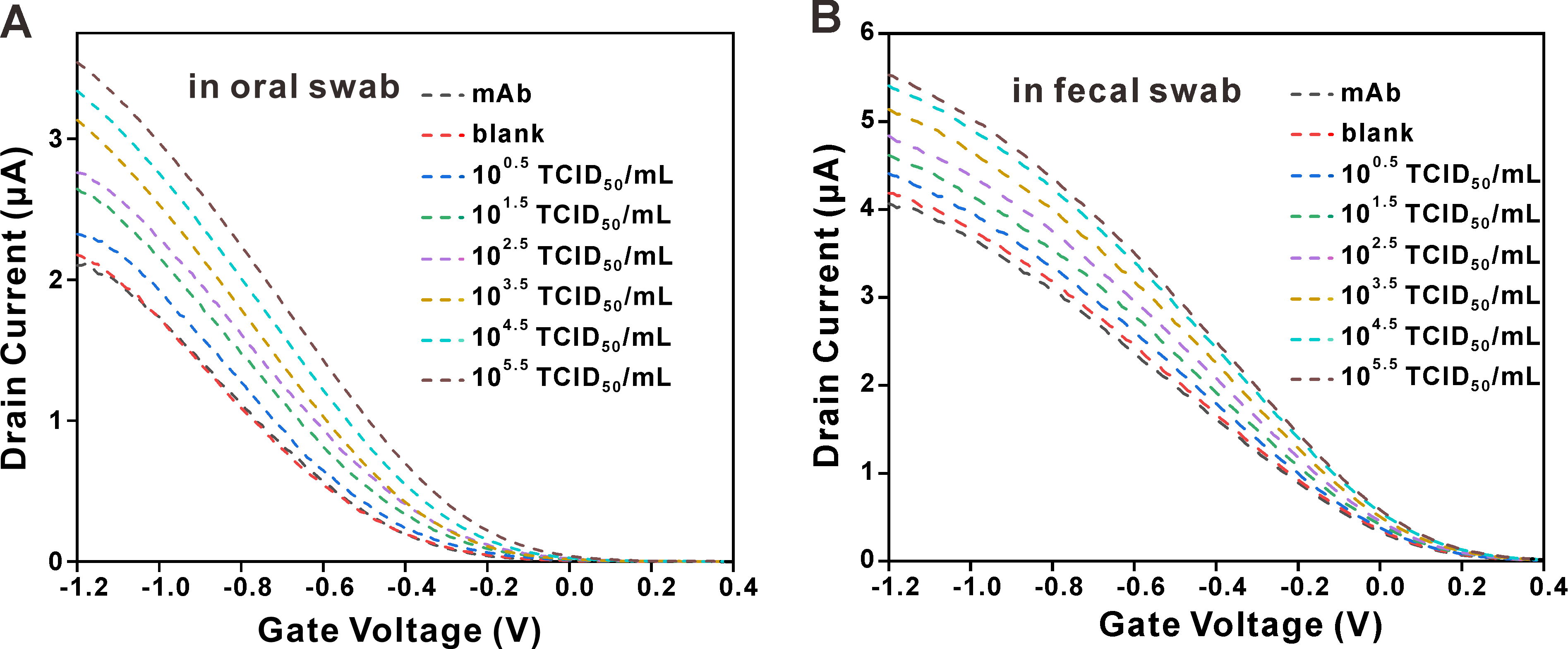


**Figure S5.** Transfer characteristic curves of sensors in oral swab (A) and fecal swab (B) for the detection of different concentrations of PEDV.


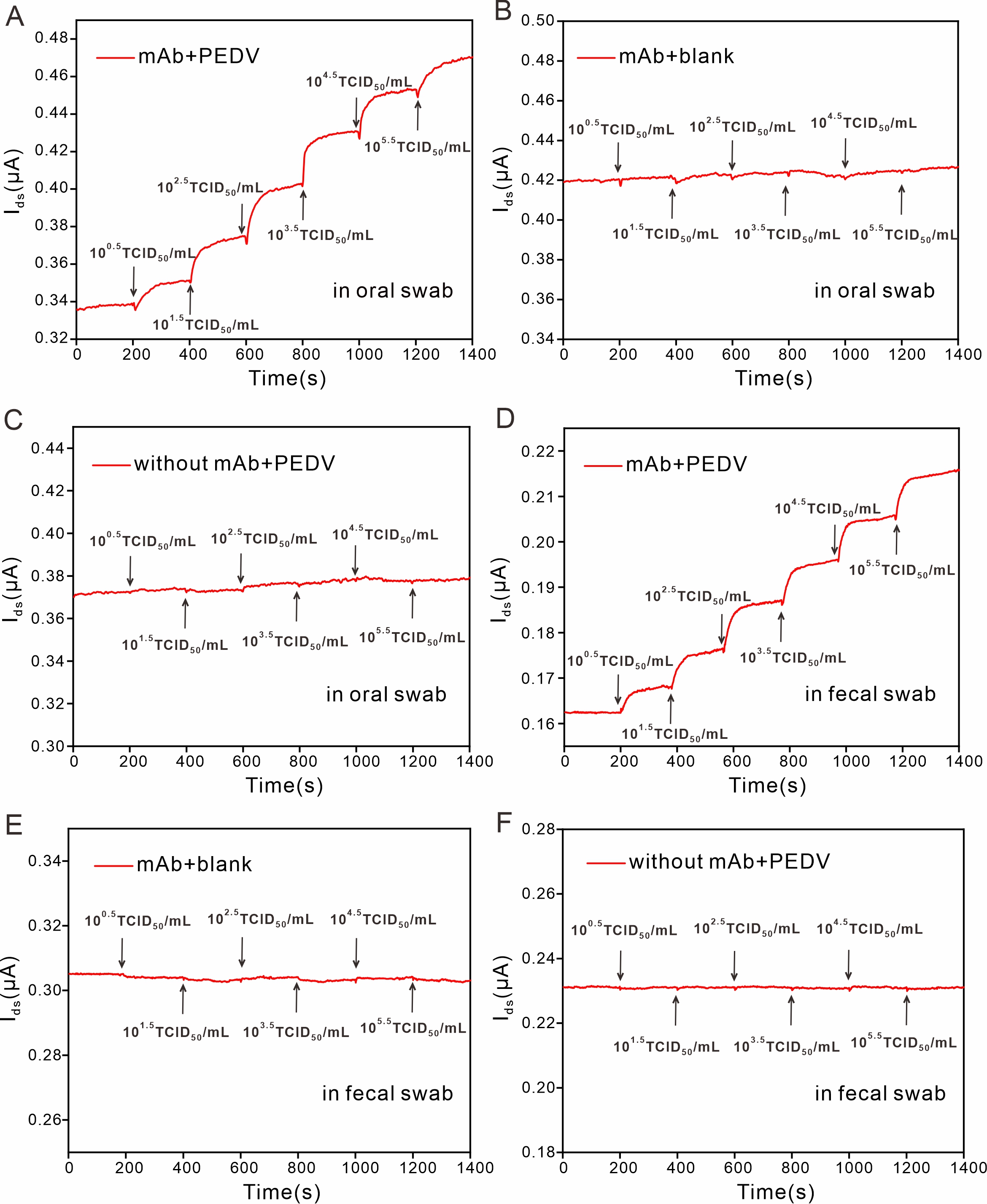


**Figure S6**. I_d_-t curves of the FG CNT-FET sensor to different concentrations of PEDV in oral swab (A-C) and fecal swab (D-E).


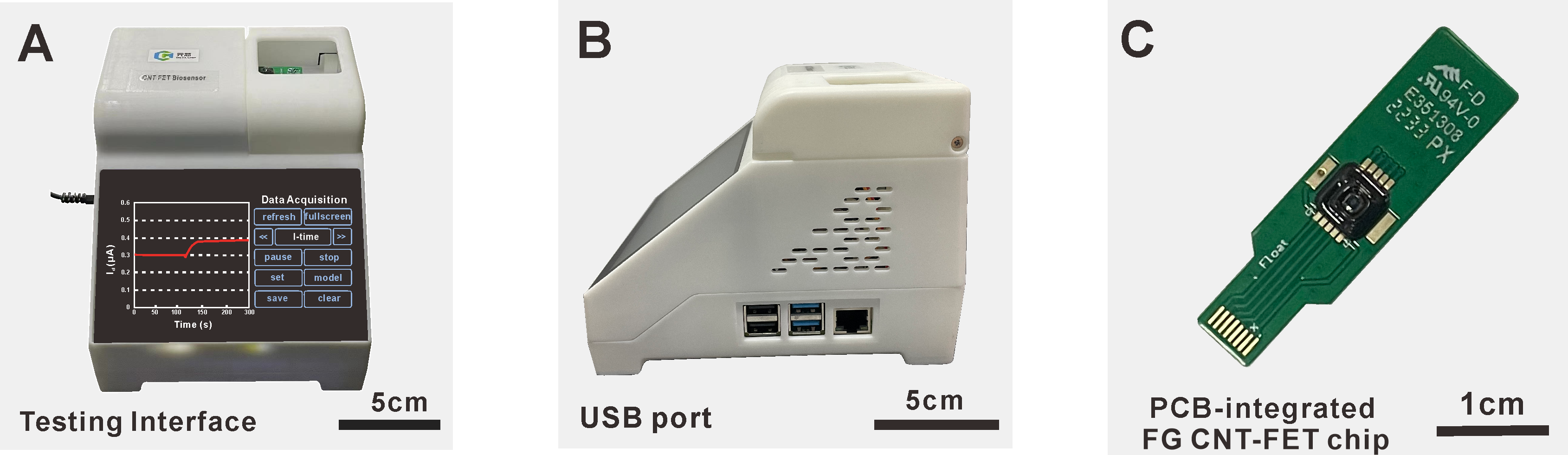


**Figure S7.** Photographs of the integrated portable device. Images of (A) the integrated portable device detection interface, (B) the USB port, and (C) the PCB-integrated FG CNT-FET chip.


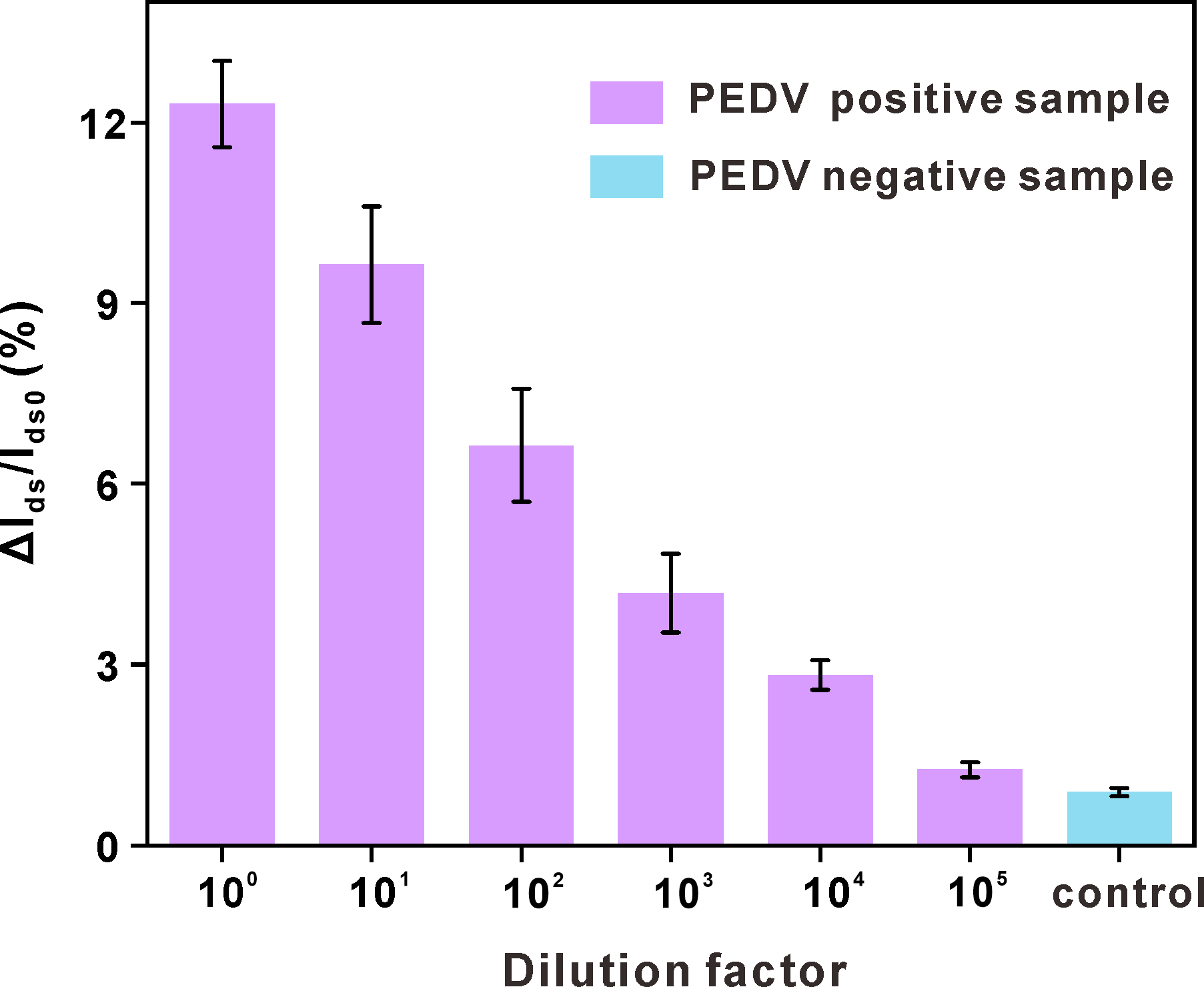


**Figure S8.** Signal response of portable devices to positive samples at different dilution factor (n = 3).


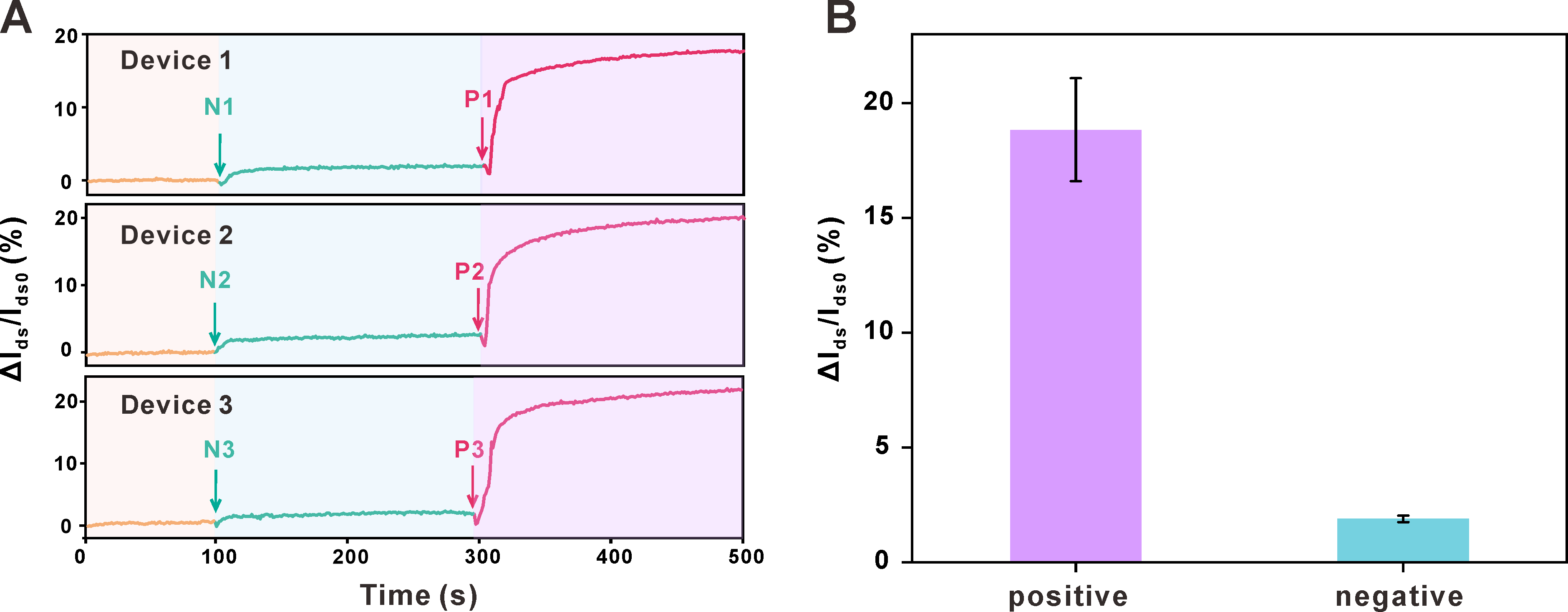


**Figure S9.** Initial validation of portable sensing platform detecting positive and negative samples. (A) Dynamic response signal of three healthy samples and three patient samples by three integrated chip. “Nn” means negative oral sample (n = 1 ~ 3), “Pn” means positive oral sample (n = 1 ~ 3). (B) Histogram of the signal response of the integrated portable FG CNT-FET sensing platform to positive and negative samples (n = 3).

**Table S1.** Comparison of the analytical performances of different methods for PEDV detection.

| **Detection method** | **Target** | **Detection limit** | **Reference** |
| --- | --- | --- | --- |
| electrochemical sensor | N-protein | 0.37 μg/mL | [1] |
| immunochromatographic assay | virus | 2.725×10^3^ TCID_50_/mL | [2] |
| lateral flow immunoassay | virus | 10^3.6^ TCID_50_/mL | [3] |
| ELISA | virus | 10^2.0^ TCID_50_/mL | [4] |
| electrochemical sensor | virus | 10^1.93^ TCID_50_/mL | [5] |
| FG CNT-FET sensor | S-protein | 8.1 fg/mL | This work |
|  | virus | 10^0.14^ TCID_50_/mL |  |

**Table S2.** Samples Ct values and double-blind testing results

| No. | Source | Ct value | qRT-PCR | Integrated portable FG CNT-FET |
| --- | --- | --- | --- | --- |
| S1 | oral swab | 30.57 | positive | positive |
| S2 | fecal swab | 20.03 | positive | positive |
| S3 | oral swab | 39.29 | negative | negative |
| S4 | oral swab | 31.55 | positive | positive |
| S5 | fecal swab | 0 | negative | negative |
| S6 | fecal swab | 29.18 | positive | positive |
| S7 | oral swab | 37.2 | negative | negative |
| S8 | fecal swab | 38.87 | negative | negative |
| S9 | fecal swab | 0 | negative | negative |
| S10 | fecal swab | 27.91 | positive | positive |
| S11 | oral swab | 28.59 | positive | positive |
| S12 | fecal swab | 39.21 | negative | negative |
| S13 | oral swab | 0 | negative | negative |
| S14 | oral swab | 26.35 | positive | positive |
| S15 | fecal swab | 38.44 | negative | negative |
| S16 | fecal swab | 0 | negative | negative |
| S17 | oral swab | 39.09 | negative | negative |
| S18 | fecal swab | 26.55 | positive | positive |
| S19 | fecal swab | 19.51 | positive | positive |
| S20 | fecal swab | 0 | negative | negative |
| S21 | fecal swab | 35.92 | negative | negative |
| S22 | oral swab | 28 | positive | positive |
| S23 | fecal swab | 22.81 | positive | positive |
| S24 | oral swab | 0 | negative | negative |
| S25 | oral swab | 24.7 | positive | positive |
| S26 | fecal swab | 29.85 | positive | positive |
| S27 | oral swab | 29.72 | positive | positive |
| S28 | fecal swab | 36.02 | negative | negative |
| S29 | fecal swab | 21.35 | positive | positive |
| S30 | oral swab | 27.57 | positive | positive |
| S31 | fecal swab | 39.27 | negative | negative |
| S32 | oral swab | 31.15 | positive | positive |
| S33 | oral swab | 0 | negative | negative |
| S34 | fecal swab | 22.48 | positive | positive |
| S35 | fecal swab | 37.76 | negative | negative |
| S36 | fecal swab | 0 | negative | negative |
| S37 | oral swab | 0 | negative | negative |
| S38 | fecal swab | 22.97 | positive | positive |
| S39 | fecal swab | 20.04 | positive | positive |
| S40 | fecal swab | 0 | negative | negative |

**References:**

1. Victorious A, Zhang Z, Chang D, Maclachlan R, Pandey R, Xia J, Gu J, Hoare T, Soleymani L, Li Y: A DNA Barcode-Based Aptasensor Enables Rapid Testing of Porcine Epidemic Diarrhea Viruses in Swine Saliva Using Electrochemical Readout. Angew Chem Int Ed Engl. 2022, 61(31): e202204252.
2. Xu F, Jin Z, Zou S, Chen C, Song Q, et al. EuNPs-mAb fluorescent probe based immunochromatographic strip for rapid and sensitive detection of porcine epidemic diarrhea virus. Talanta 2020, 214:120865.
3. Zou S, Wu L, Li G, Wang J, Cao D, Xu T, Jia A, et al. Development of an Accurate Lateral Flow Immunoassay for PEDV Detection in Swine Fecal Samples with a Filter Pad Design. Anim Dis. 2021, 1(1):27.

4. Fan B, Sun J, Zhu L, Zhou J, Zhao Y, Yu Z, Sun B, Guo R, He K, Li B. Development of a Novel Double Antibody Sandwich Quantitative Enzyme-Linked Immunosorbent Assay for Detection of Porcine Epidemic Diarrhea Virus Antigen. Front Vet Sci. 2020, 7:540248.

5. Li Z, Luo Y, Huang Z, Zhao C, Chen H, El-Ashram S, Huang J, Su L, Zhang W, Ma G, et al. An ultrasensitive electrochemical sensor for detecting porcine epidemic diarrhea virus based on a Prussian blue-reduced graphene oxide modified glassy carbon electrode. Anal Biochem. 2023, 662:115013.
